# Pain of methadone-maintained heroin addicts: lonelier individuals feel more intense pain

**DOI:** 10.18632/oncotarget.20387

**Published:** 2017-08-22

**Authors:** Fu Li, Yan-Min Xu, Jun-Hong Zhu, Jin Lu, Bao-Liang Zhong

**Affiliations:** ^1^ Affiliated Wuhan Mental Health Center (The Ninth Clinical School), Tongji Medical College of Huazhong University of Science and Technology, Wuhan, Hubei Province, China; ^2^ Department of Psychiatry, The First Affiliated Hospital of Kunming Medical University, Kunming, Yunnan Province, China

**Keywords:** pain, loneliness, heroin addiction, methadone

## Abstract

Managing pain in patients with heroin addiction is challenging, because most pain medications are addictive. A promising way to relieve pain of heroin addicts is to identify and address modifiable psychosocial factors associated with pain. This study examined the association between loneliness and pain intensity in Chinese heroin addicts receiving methadone maintenance treatment (MMT). A consecutive sample of 603 heroin addicts were recruited from three MMT clinics in Wuhan, China. Loneliness was assessed with a single question, and socio-demographic and clinical data were collected with a standardized questionnaire. Pain intensity was assessed with the five-point Verbal Rating Scale. Multiple ordinary logistic regression was used to control for potential confounders that may bias the loneliness-pain relationship. There was a significant and positive correlation between pain intensity and loneliness scores among methadone-maintained heroin addicts (*r* = 0.453, *P* < 0.001). After controlling for potential socio-demographic and clinical confounders, an increase in the level of loneliness was significantly associated with an increase in pain intensity (OR = 1.22, *P* = 0.042). Loneliness is significantly associated with pain of methadone-maintained heroin addicts. Psychosocial interventions aimed at reducing loneliness might prevent or reduce pain of patients receiving MMT.

## INTRODUCTION

Pain is a common health issue in patients with opioid use disorders [1, 2]. In China, studies have found a 39.7% prevalence of chronic severe pain in heroin addicts from compulsory detoxification centers and a 14.1% prevalence of clinically significant pain in heroin addicts from methadone maintenance treatment (MMT) clinics [3, 4]. The presence of pain is significantly associated with reduced quality of life, poor response to MMT for opioid dependence, and opioid addiction relapse of heroin addicts [3, 5, 6]. Therefore, effective pain management is very important in addiction treatment practice. However, managing pain in heroin addicts is particularly challenging, because most pain medications are addictive [7].

In China, due to institutional exclusion and social stigma towards individuals with heroin addiction, heroin addicts often have poor social support networks and are socially isolated [8, 9]. Because social isolated persons are more likely to feel lonely [10, 11], loneliness might be a prevalent psychosocial problem in heroin addicts.

Psychosocial factors also play an important role in the etiology, clinical course, and prognosis of pain [12]. Studies with samples of adolescents, cancer survivors, caregivers of dementia patients, and fibromyalgia patients have consistently reported loneliness as a significant predictor of pain [13–15]. Evidence from systematic reviews shows that loneliness can be relieved by a range of interventions [16, 17]. Therefore, identifying and addressing modifiable psychosocial factors associated with pain such as loneliness, would be a promising way to reduce pain and its associated negative outcomes. However, in heroin addicts, it is unclear that whether loneliness is significantly associated with their pain.

China has the largest population of drug users globally. In 2004, China initiated its MMT program, which, until now, has been the largest MMT program in the world [18, 19]. By 2014, there had been a total of 765 community-based MMT clinics providing services to 0.19 million illicit drug (mainly heroin) users in 28 provinces of the mainland China [20]. Given the lack of empirical studies on loneliness-pain link in heroin addicts and the quite large number of methadone-maintained patients in China, the present study examined the association between loneliness and pain intensity in Chinese heroin addicts of MMT clinics.

## RESULTS

In total, 603 patients completed the survey. The average age of the 603 patients was 38.1 years (standard deviation [SD] = 7.0), and 68.3% were males. Scores of Zung’s Self-rating Depression Scale (SDS) and Zung’s Self-rating Anxiety Scale (SAS) were 38.6 (SD = 8.4) and 44.8 (SD = 9.6), respectively. Detailed socio-demographic and clinical characteristics of the study participants are displayed in Table 1.

**Table 1:** Multiple ordinal logistic regression analysis on the effect of loneliness on pain intensity

Variable		*n*	Pain Intensity Score				
			Standardized Coefficient	Standard Error	Wald χ^2^	OR (95% CI)	*P*
Loneliness score		603	0.196	0.097	4.14	1.22 (1.01,1.47)	0.042
Gender	Male	412	0.045	0.188	0.057	1.05 (0.72,1.51)	0.812
	Female	191	1				
Age(year)#	< 39	300	−0.071	0.192	0.137	0.93 (0.64,1.36)	0.712
	≥ 39	303	1				
Length of Education#	< 9	140	−0.785	0.209	14.129	0.46 (0.30,0.69)	< 0.001
	≥ 9	463	1				
Marital Status	Never-married	154	0.324	0.215	2.26	1.38 (0.91,2.11)	0.133
	Others*	154	0.154	0.235	0.431	1.17 (0.74,1.85)	0.512
	Married	295	1				
Route of past heroin use	Injection	507	0.003	0.233	0.001	1.00 (0.64,1.58)	0.991
	Smoking	96	1				
Duration of past heroin use (year)#	< 10	230	−0.237	0.181	1.721	0.79 (0.55,1.12)	0.190
	≥ 10	373	1				
MMT dose (mg/d)#	< 70	273	−0.324	0.178	3.318	0.72 (0.51,1.03)	0.069
	≥ 70	330	1				
MMT duration (months)#	< 24	240	0.578	0.185	9.811	1.78 (1.24,2.56)	0.002
	≥ 24	363	1				
Depressive symptoms	Yes	204	0.939	0.212	19.659	2.56 (1.69,3.87)	< 0.001
	No	399	1				
Anxiety symptoms	Yes	219	1.404	0.246	15.454	4.07 (2.51,6.59)	< 0.001
	No	384	1				

#Continuous variables were dichotomized via the median split procedure.

*Others include remarried, divorced, separated, and widowed.

Scores of loneliness and pain intensity score of methadone-maintained heroin addicts were 2.9 (SD = 1.0) and 2.8 (SD = 1.1), respectively. Loneliness and pain intensity were significantly and positively correlated (*r* = 0.453, *P* < 0.001).

After controlling for the confounding effects of socio-demographic and clinical variables with multiple ordinary logistic regression, an increase in the level of loneliness was significantly associated with an increase in pain intensity (OR = 1.22, *P* = 0.042).

## DISCUSSION

Heroin addiction is not only a medical issue but also a social issue, thus the clinical management of any health problem in heroin addicts should take psychosocial factors into consideration. The present study determined the relationship between loneliness and pain in patients of MMT clinics, and found a statistically significant moderate, positive correlation between the level of loneliness and pain intensity. After controlling for the potential confounding effects of other covariates, loneliness remained significantly associated with pain, suggesting an independent effect of loneliness on pain in heroin addicts.

Most existing studies on the health consequences of loneliness focused on middle-aged and older adults, showing the significant contribution of loneliness to increased mortality, metabolic syndrome, depression, and reduced cognitive functions of this population [10, 21–23]. Findings from our study suggest that loneliness is also a significant contributor to increased pain intensity. Pain is conceived as not only an unpleasant sensory but also an emotional state, so the complex process of pain is subject to a variety of psychosocial factors [24, 25]. For example, the study by Wolf and colleagues found lonely episodes were associated with subsequent increases in negative thinking about pain, which in turn exemplified the perception on pain intensity of a stimulus [15]. Therefore, the significant loneliness-pain link revealed in this study may be explained by the increased negative cognition on pain resulted from loneliness. In addition, loneliness (in particular chronic loneliness) can suppress the functions of the immune system, making lonely individuals susceptible to a high inflammation status [26]. There is evidence that pain or hyperpathia can be caused by a variety of bioactive compounds released by inflammatory cells such as neurokinin, bradykinin, histamine, and inflammatory cytokines [27]. This is another possible biological mechanism underlying the loneliness-pain association.

This study has a few limitations. First, data of this study were collected cross-sectionally, which limits the ability to make causal inference on the loneliness-pain relationship. Second, due to deficiencies in the study design, we did not collect data on possible inflammatory cytokines and other bioactive compounds to explore the biological mechanism of how loneliness impacts pain. Third, due to the stigma associated with loneliness, the direct measure of loneliness used in this study, the single-item question, may result in an underestimate of the actual prevalence of loneliness [28]. As a result of this, our study might underestimate the loneliness-pain association.

Despite the limitations of the study, this study reveals that loneliness is significantly associated with pain in Chinese heroin addicts of MMT clinics. The research results suggest that providing psychosocial interventions such as those enhancing social support and addressing maladaptive social cognition [16] cannot only reduce loneliness, but also potentially reduce the pain of patients in MMT clinics.

## MATERIALS AND METHODS

### Subjects

This is a secondary data analysis based on a cross-sectional survey conducted in three city-owned MMT clinics in Wuhan, China, between June 2009 and July 2010 [3, 29, 30]. The study consecutively enrolled 652 adult heroin addicts who met DSM-IV criteria for a lifetime diagnosis of heroin addiction. Patients with severe physical diseases, alcohol dependence, organic mental disorders, or psychotic symptoms, were excluded from the survey.

The Ethics Committee of Wuhan Mental Health Center approved the study protocol prior to the formal study. All participants provided written informed consent.

### Procedures and assessments

This study was a self-administered questionnaire survey. Trained psychiatrists working in MMT clinics were arranged to read out questions for subjects who were illiterate or had difficulties in reading. Before the collection of these questionnaires, our investigators checked the completeness and coherence of responses to questions in the questionnaire.

A questionnaire was specifically designed for this study. Because previous population-based studies have reported a significant associations between socio-demographic factors, particularly low socio-economic status (as measured by education and income), and pain [31–33], socio-demographic variables in the questionnaire included gender, age, education years, marital status, employment status, and self-rated financial status (good, fair, poor). Because substance use characteristics (i.e., long-term heroin use and injection drug use) and psychological factors have been found to be significantly associated with pain [34–36], clinical data collected in the study were usual route of past heroin administration (smoking, injecting), duration of past heroin use, duration of MMT, methadone dosage, depression, and anxiety. Depression and anxiety were assessed with SDS and SAS, respectively [37]. The total scores of both SDS and SAS vary between 20 and 80, and a cut-off scores of 40 or higher in Chinese SDS and 43 or higher in Chinese SAS are used to denote clinically significant depressive and anxiety symptoms, respectively [37].

Loneliness was measured with a single-item self-report question “How often do you feel lonely?” with a five-point scale: 1=never, 2=seldom, 3=sometimes, 4=often, 5=always. This single-item measure of loneliness is widely used in previous studies and it has good validity in assessing the level of loneliness [10, 11, 28, 38–43]. Compared multi-item loneliness scales (i.e., the UCLA Loneliness Scale), this single-item measure of loneliness is time-saving and can reduce subjects’ response burden, which are particularly suitable for subjects with low level of educational attainment. Importantly, this single item does avoid potential confounding that might stem from multi-item loneliness measures [10], for example, the UCLA Loneliness Scale uses social support to represent loneliness indirectly.

Pain intensity was assessed with the five-point Verbal Rating Scale (VRS), asking “Overall, how intense is your pain now?”, with a five-category responses: 1=none, 2=mild, 3=moderate, 4=severe, 5=very severe. This five-point VRS measure of pain has been widely adopted in prior studies and studies have shown that its validity was as good as other common measures of pain intensity [44, 45].

### Statistical analysis

Loneliness and pain intensity scores of heroin addicts were described, and Pearson Correlation Coefficient was used to measure the strength of the association between the two. The impact of loneliness on pain was examined with multiple ordinary logistic regression that entered pain intensity score as the outcome variable, loneliness score as the predictor, and socio-demographic and clinical covariates at once to adjust for the potential confounding effects of these socio-demographic and clinical variables. The statistical significance level was set at *p* < 0.05 (two-sided). SPSS software version 20.0 package was used for analyses.
